# Environmental filtering and spillover explain multi-species edge responses across agricultural boundaries in a biosphere reserve

**DOI:** 10.1038/s41598-020-71724-1

**Published:** 2020-09-09

**Authors:** J. van Schalkwyk, J. S. Pryke, M. J. Samways, R. Gaigher

**Affiliations:** Department of Conservation Ecology and Entomology, Stellenbosch University, Private Bag X1, Matieland, 7602 South Africa

**Keywords:** Ecology, Agroecology, Biodiversity, Community ecology, Conservation biology

## Abstract

To ensure integrity of protected areas we need to understand how species respond to anthropogenic borders. We investigate, from a metacommunity perspective, the direct and indirect mechanisms by which transformed areas affect distribution patterns of ground-living arthropod assemblages inhabiting an extensive protected area adjacent to fruit orchards in an important biosphere reserve. Arthropods and environmental variables were sampled along transects perpendicular to natural-orchard edges. Influence of distance from orchard boundary, degree of impermeability of the boundary, orchard habitat quality (local scale land-use intensity), and edge-induced changes in local environmental variables on arthropod species richness and composition in non-crop habitats were assessed. Arthropod groups were assessed in terms of habitat fidelity: species associated with natural habitat (stenotopic species), those within crop habitat (cultural species), and those showing no preference for either habitat (ubiquitous species). Spillover resulted in higher cultural species richness near edges, but not higher overall species richness. Environmental filtering was important for stenotopic species composition, which was influenced by edge-induced changes in environmental variables. Ubiquitous species composition was determined by orchard impermeability. Increased orchard habitat quality was associated with higher cultural and ubiquitous species richness. The effects of orchards on assemblages in natural habitats can be variable, but predictable when using species habitat specificity in conjunction with a metacommunity framework. High intensity orchards may act as sink habitats, especially for species that readily disperse between crop and natural habitats. Here we recommend that local buffer strips are > 85 m wide, which will reduce the influence of cultural species spillover on sensitive natural ecosystems.

## Introduction

Protected areas are not closed systems and are affected by land-use change outside their borders, therefore managing these areas in isolation makes their long-term sustainability uncertain^1^. Sustainable conservation requires a landscape approach that considers the larger geographical area in terms of both social and ecological systems, as well as their interactions^2^. The United Nations Education, Scientific and Cultural Organization’s (UNESCOs) Man and the Biosphere Programme is considered one of the better options for integrating conservation with surrounding landscapes, as it recognizes that landscapes can be transformed along a gradient of land-use intensity. In this regard, Biosphere Reserve (BR) buffer and transition zones are key functional spaces that represent the interface between conservation and resource extraction.

To effectively manage and design these spaces, it is critical to understand how species respond at boundaries between transformed areas and adjacent protected areas^3,4^. There are three main ways that transformed areas can influence species in natural areas: (1) through movement and dispersal, (2) resource availability, and (3) through the abiotic environment^5,6^. Movement between transformed and natural areas is greater when they are structurally more similar^7,8^. When transformed areas are supplementary or alternative sources of essential resources, they can influence populations in the adjacent natural areas^6,9,10^. Edges (i.e. boundary between two ecosystem types) can cause spatial variation in biologically important abiotic variables, and influence ecological responses through indirect pathways mediated by local conditions^11^. For example, distance from the edge influences biotope structure^12–14^, and unique species composition at edges can result from the blending of juxtaposed environmental conditions^15^.

These three main effects do not operate in isolation. For example, how species are able to establish (and have access to resources) in transformed areas may be dependent on levels of contrast across boundaries^8^, while the quality of transformed areas can lead to different edge and isolation effects^16,17^. Intrinsic characteristics related to species’ traits will also influence the effect of transformed areas^5^. A species’ degree of habitat specialization can influence its perception of habitat size and isolation^18^. For example, among butterflies, specialists are less likely to move from natural areas into transformed areas than are generalists^19^. A species’ degree of habitat specificity can also determine if and for how long it is exposed to management practices in transformed areas^20,21^.

Edge studies have largely focused on species-level responses^5,22^. Multi-species responses (i.e. changes in species richness and composition) to edges may be explained when patterns are incorporated into current theory of community assembly^6,23^. Metacommunity theory describes processes that occur at the scale of the metacommunity, i.e. a set of local communities that are linked by dispersal of multiple, potentially interactive species^18,24^. Community responses at edges can be viewed as the unique product of spatio-temporal interaction between patch context and landscape context^5,23,25,26^, and as the metacommunity concept provides a way of thinking about the interplay between local environmental and regional processes in structuring local communities^18,27^, it provides an important framework for investigating assemblage level responses at edges.

At the community level, specialization has been linked to the relative importance of species sorting^18^, with local environmental variables being more important for specialists than generalists^27,28^. This suggests that specialists may be particularly sensitive to edge-induced changes in the local abiotic environment. Source-sink dynamics also play a role, and enable mixing of species at edges through mixing of immigrants from adjacent habitats (i.e. mass effects or spillover)^29,30^. In this case, high dispersal rates maintain species in sites with negative growth rates^31^ and override the effects of local selection^8,18,30,32^.

In crop systems, we can distinguish three broad groups of species based on their spatial distribution. Firstly, stenotopic species are predominantly in non-crop areas and rarely occupy crop fields. Secondly, cultural species occur mostly in crop fields, and thirdly, ubiquitous species occur in both crop and non-crop areas while showing no preference for either^33^. On this basis, we investigate here, from a metacommunity perspective, the mechanisms that differentially affect stenotopic, cultural, and ubiquitous epigaeic (ground-living) arthropod species in non-crop areas near the edges of agricultural areas in a highly diverse protected area.

A metacommunity framework can improve our understanding of the patterns of biological communities along natural-anthropogenic interfaces in BRs. Here we assess the value of such a framework for predicting variation in arthropod assemblages in natural areas adjacent to deciduous fruit orchards in a BR. We assess the influence of distance from orchard boundary, orchard impermeability, orchard habitat quality, and edge-induced changes in local environmental conditions on arthropod species richness and composition in adjacent non-crop habitats. We hypothesize that transformed areas will differentially affect each of the three groups (stenotopic, cultural, and ubiquitous). Since local environmental variables are more important for specialists^27,28^, we expect stenotopic species to be affected more by edge-induced changes in local environmental conditions in natural areas. In turn, we hypothesize that cultural and ubiquitous species in natural areas will be strongly influenced by characteristics of the agricultural area. Specifically, we expect higher diversity of these groups in natural areas adjacent to orchards with higher habitat quality^34^. We also expect cultural species diversity to show a decline with distance from the crop edge in the form of spillover attrition^35^. Furthermore, we expect impermeability to be more important for ubiquitous species in natural areas, as this would determine their exposure to orchard habitat quality.

## Methods

### Study area and design

**Figure 1:**
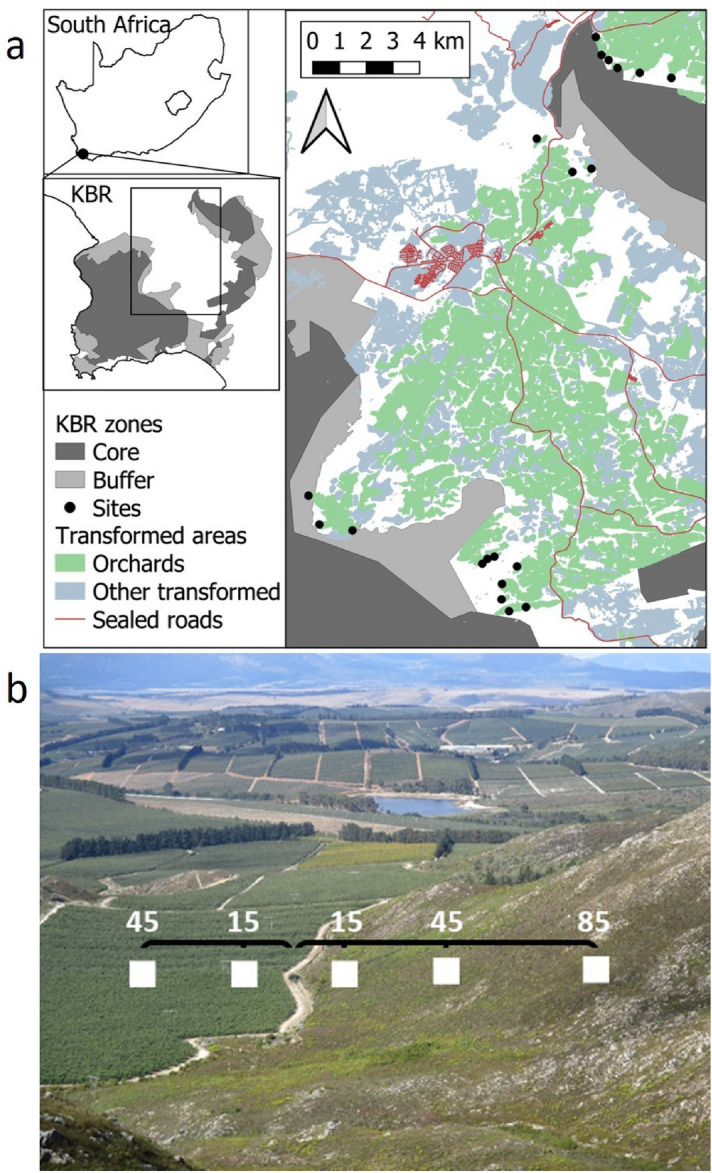
**a** Map of study sites across the Kogelberg Biosphere Reserve (KBR), and **b** position of plots across the orchard-fynbos interface. Distances that plots were positioned from edge are shown in meters. Maps were generated using QGIS 3.12.1 (https://www.qgis.org/).

We sampled in the Kogelberg Biosphere Reserve (KBR), on the western edge of the Cape Floristic Region biodiversity hotspot in south-western South Africa (Fig. 1a). The dominant vegetation of our sampling area is fynbos, a schlerophyllous vegetation type adapted to low soil nutrients, winter rainfall, and regular fires. Deciduous pome fruit orchards dominate this landscape. Most of the fruit produced is for export^36^, and the orchards are characterized by highly specialised conventional management^37^. Twenty sites were selected across the KBR that represent the boundaries between extensive natural areas (fynbos) and deciduous fruit orchards (Fig. 1a). Farmers provided information on application of insecticides, fertilizers, and cover crop management. All orchards were subject to integrated pest management (details in Tables S1 and S2). Orchards were 4–37 years old, and distances between sites were 300–21 000 m.

### Sampling

Epigaeic arthropods were sampled over two seasons: spring (October) 2015 and autumn (March) 2016, coinciding with flowering and fruiting stages of the crop trees. Each site consisted of three plots positioned in the fynbos at increasing distances from the nearest orchard edge: 15 m, 45 m and 85 m (Fig. 1b). Additional plots were placed in the adjacent orchard at 15 m and 45 m from the orchard-fynbos interface. These latter orchard plots were only used in subsequent analyses to classify species according to their habitat associations, and the focus of this study is on the plots in the fynbos. Each plot consisted of four pitfall traps, arranged as a 10 × 10 m square.

Each pitfall trap was 7 mm in diameter, filled with a 50% ethylene glycol solution, and left open for 5 days per season (10 days in total). Sampled arthropods were sorted to genus, family, or superfamily (except mites, most of which could only be sorted to order), and then to morphospecies (referred to as ‘species’ from now on). Plot level species data consisted of data from the four pitfall traps pooled across both sampling seasons.

### Environmental variables

The effect of orchards on adjacent natural areas were represented by four variables: (1) distance from edge, (2) edge related changes in local environmental conditions, (3) impermeability, and (4) land-use intensity (Table 1; more details on each measure are provided in Appendix 1). We also refer to these variables as ‘edge-related variables’.

**Table 1:** Edge related variables included in the analyses of assemblages in fynbos adjacent to orchards.

Variable	Description
Distance from edge	Distance from orchard edge boundary into fynbos. Continuous and measured in meters
Edge related change in local environmental conditions	Variability in local environmental conditions in fynbos related to edge effects. Measured as the principal component of DAPC. Higher values indicate more edge-related change in local environmental conditions. Continuous and unitless
Impermeability	Composite measure that describes amount of contrast of adjacent orchard. Higher values indicate lower permeability. Continuous and unitless
Local land-use intensity	Compound index that summarized standardized intensity of pesticide application, cover crop management, and fertilization in adjacent orchards. Continuous and unitless

*DAPC* discriminant analysis of principal components.

Local environmental variables measured for each plot were vegetation structure and soil moisture content (Table A1). To reduce the number of variables that describe edge-related changes in local environmental variables in fynbos, we used Discriminant Analysis of Principal Components (DAPC) to describe diversity in the local environmental variables of pre-defined groups (i.e. each of the distance categories in fynbos). We then used the principal component of DAPC which showed the strongest discrimination between distance classes (15 m, 45 m and 85 m in fynbos) to describe edge-related changes in local environmental variables. We used DAPC instead of other commonly used data reduction methods because we were not interested in variability between plots across all sites, which would have been influenced by differences in fire history among sites. Rather, we focused on differences in local between-group variability among the various distance classes.

Impermeability is a composite measure that describes amount of contrast between orchards and adjacent fynbos (higher values indicate less permeability) (Table A2). It incorporates orchard age, and the orientation of both orchard edge (crop to natural area interface) and tree rows relative to sun position. Land-use intensity was characterized using a quantitative, continuous index based on farmers’ information (measures of agrochemical input and cover crop management), and measurements of understorey plant family richness in each orchard (Table A3).

While interest here lay in the influence of orchards on arthropod assemblages in natural habitats, we also need to account for changes in assemblage structure that are due to variation in unrelated environmental variables (i.e. background environmental heterogeneity). Variables describing background environmental heterogeneity were selected based on previous exploratory analyses^38^. These consisted of both site-measured variables, as well as variables derived from spatial layers, and included information on fire history, geology, meso-climate, and local biotope variables (details in Table S3).

### Data analyses

To measure the effectiveness of sampling effort, species accumulation curves were plotted for fynbos and orchard plots respectively. Species richness was estimated using the Chao estimator^39^. These analyses used the package *vegan*^40^ in R, version 3.6.0^41^.

#### Species richness

Species were categorised as stenotopic, cultural, or ubiquitous, based on their preference for one or both ecosystems, i.e. only in the natural fynbos area (stenotopic species), only in the orchard (cultural species), or present in either fynbos or orchard (ubiquitous species). Habitat specificity was calculated using Pearson’s phi coefficient of association using the package *indicspecies*^42^ in R. Species were classified as habitat specialists when probability of association was < 0.05, after correcting for unequal sample sizes^43^. Species not showing strong association with either fynbos or orchard were classed ubiquitous. Since it is not practically possible to determine habitat preferences of singleton species, all analyses conducted on ubiquitous species were repeated with singletons removed.

All analyses were conducted on observed species richness. Regression models were used to determine influence of orchards on species richness in natural fynbos at each plot (15, 45, and 85 m from orchard edge, n = 60). Explanatory variables included in the models were distance to orchard edge, edge-related change in local environmental variables (DAPC scores), impermeability, and land-use intensity. Generalized linear mixed-effects models (GLMMs), with a Poisson distribution and site as a random effect, were used to evaluate variables of interest (fixed effects). Interaction terms were not included, as it made the model too complex considering the sample size. We used adaptive Gauss-Hermite quadrature to estimate GLMM parameters. The data did not exhibit over-dispersion, and significance of fixed effects was based on Type II Wald Chi-square tests. The above procedure was repeated for all species, all species with cultural species excluded, stenotopic species, cultural species, and ubiquitous species, as well as for ubiquitous species with singletons removed. GLMMs were performed with the package *lme4*^44^ in R.

#### Species composition

To relate assemblage patterns to edge influences, we used canonical ordination to estimate how much variation in species composition in natural fynbos could be explained by edge-related variables after accounting for background environmental heterogeneity and residual spatial autocorrelation. We used transformation based redundancy analysis (RDA), and selected Hellinger transformation, as it yielded the highest fraction of explained variance^45^. To partition the variation in assemblage structures, we used partial RDA. Previous analyses suggest importance of climate, fire history, and geological variables for ground arthropod diversity^38^, and these variables were included as representative of background environmental heterogeneity (Table S3). We used a global test of significance, and only submitted the variables representing background environmental heterogeneity to forward selection, when this was significant. We used Moran’s eigenvector maps (MEMs) to describe spatial structures^46^. To detect spatial patterns in the residuals once effect of environmental predictors had been removed, we used residuals of the model fitted with edge variables (and variables representing background heterogeneity when significant), and used forward selection with double stopping criteria to select significant MEM variables^47,48^. This was repeated for several candidate spatial weighting matrices^48^. In the final partial RDA, we partitioned out variance, due to background environmental heterogeneity and spatial structures, to quantify variation explained by distance-to-edge, edge-induced changes in local environmental variables, impermeability, and land-use intensity. For each RDA model, we performed permutation tests for the spatial independence of residuals to check for significant spatial autocorrelation at short lag distances^49^. We performed separate significance tests for each marginal term in the model with all other terms, and used partial RDA to isolate the effect of each explanatory variable. The above procedure was repeated for all, all with cultural species excluded, stenotopic, cultural, and ubiquitous species, as well as for ubiquitous species with singletons removed. Partial RDAs were performed using the *vegan* package and MEMs constructed and selected using the *adespatial* package^50^ in R.

## Results

### Species richness

Overall, despite sampling 20 573 individuals from 434 species, species accumulation curves did not reach asymptotes (Appendix 2) and the Chao-estimated total species richness was 636.57 (± 43.75) species. The most species-rich groups overall were beetles (102 species), mites (84 species) and spiders (74 species). The most species-rich groups in fynbos were also beetles (83 species), mites (72 species) and spiders (69 species). Species diversity was higher in natural fynbos than in orchards. In fynbos, most species were ubiquitous (297 species, 135 excluding singletons), followed by stenotopic species (60 species), and then cultural species (32 species).Table S4 lists all sampled species and their abundances.

**Figure 2:**
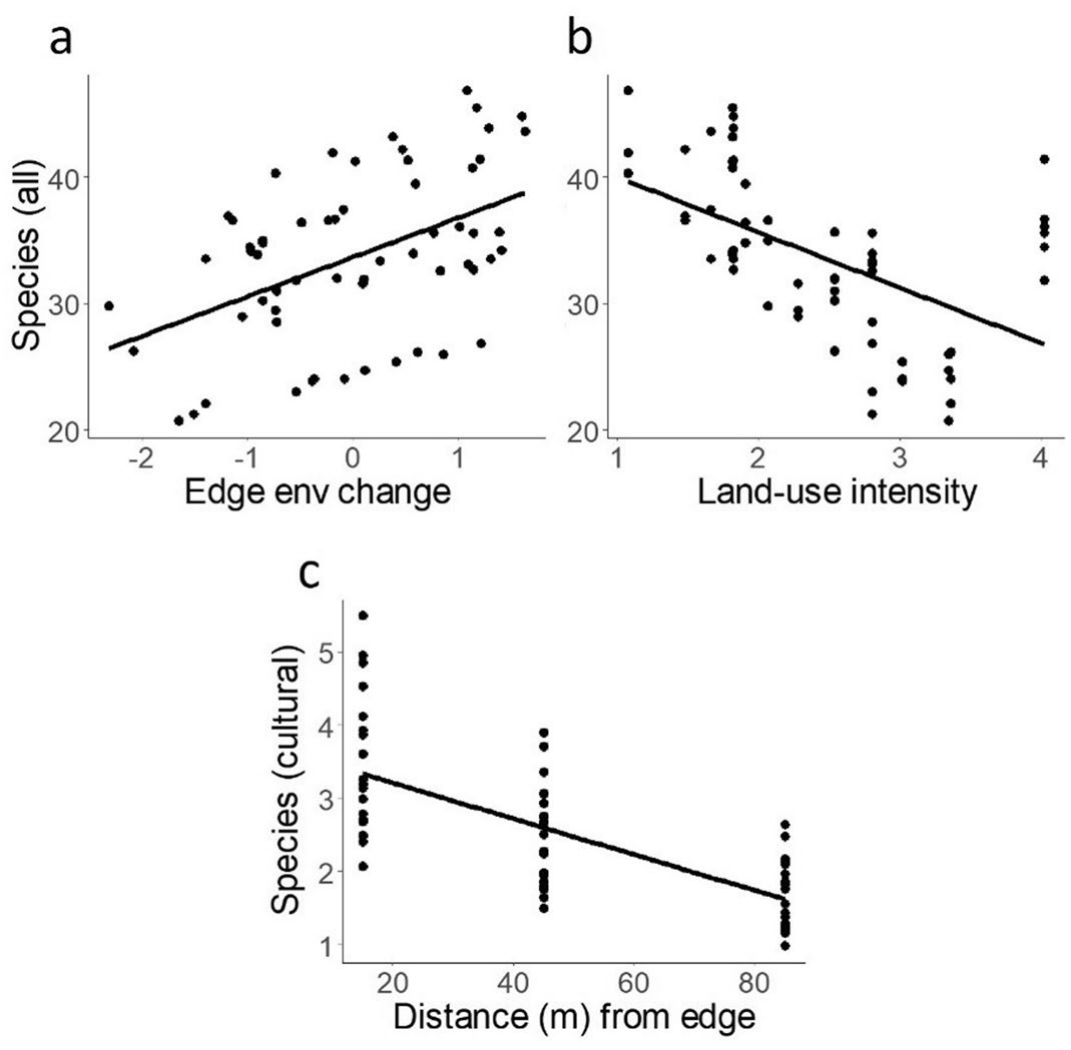
Generalized linear mixed model relating predicted species richness of all species in fynbos to **a** edge related changes in local environmental variables (principal component of Discriminant Analysis of Principal Components) an **b** land-use intensity in adjacent orchards, as well as cultural species in fynbos to **c** distance from orchard edge.

Results from GLMMs showed that edge-associated change in local environmental variables was important for all species and ubiquitous species richness (Table 2, Fig. 2a and Figure S1). This was also the case for ubiquitous species with singletons removed (Figure S1). Edge-associated change in local conditions was negatively correlated with distance-to-edge, and higher species richness was associated with changes in local environmental conditions close to the orchard edge. None of the measured predictors were important for stenotopic species. Land-use intensity was important for all species, cultural species, ubiquitous species, and ubiquitous species with singletons removed, and showed a negative relationship with species richness (Fig. 2b and Figure S1). Distance-to-edge was only important for cultural species richness, and showed a negative relationship with cultural species richness (Fig. 2c).

**Table 2:** Results of generalized linear mixed-effects models showing the effects of distance from edge, edge associated changes in local environmental variables, impermeability and local land-use intensity (LUI) on species richness of all, stenotopic, cultural, and ubiquitous species in fynbos.

	Distance edge	Edge. env	Impermeability	LUI
**All species (R**^**2**^** = 0.62)**				
Chi-sq	0.08	7.14**	0.01	5.74*
Est	0.01	0.09	− 0.01	− 0.13
**All min cultural (R**^**2**^** = 0.57)**				
Chi-sq	1.17	7.99**	0.01	5.07*
Est	0.03	0.10	− 0.01	− 0.12
**Stenotopic (R**^**2**^** = 0.40)**				
Chi-sq	2.65	2.52	0.01	1.12
Est	0.06	0.07	0.03	− 0.08
**Cultural (R**^**2**^** = 0.33)**				
Chi-sq	6.51*	0.25	0.11	5.13*
Est	− 0.26	0.05	− 0.03	− 0.28
**Ubiquitous (R**^**2**^** = 0.43)**				
Chi-sq	0.01	7.65**	0.02	9.27**
Est	0.01	0.14	− 0.04	− 0.16
**Ubiquitous min singletons (R**^**2**^** = 0.42)**				
Chi-sq	0.01	6.92**	0.02	5.65*
Est	0.01	0.16	− 0.01	− 0.15

Site was included as a random variable in all models. Values represent Wald-chi squared values. All min cultural—all species excluding cultural species. Significant results (*p* < 0.05) are shown in bold. Distance edge—distance from orchard edge, Edge. env—edge-associated change in local environmental variables, LUI—land-use intensity. R^2^—conditional coefficient of determination.

*p < 0.05; **p < 0.01.

### Species composition

Variables related to background environmental heterogeneity were important for all groups, except cultural species (Table S5). When including selected background environmental variables along with edge-related variables, none of the model residuals showed significant spatial patterns. None of the partial RDA therefore contained MEMs as spatial predictors. Final partial RDAs indicated that edge-related variables explained 8.71% of variation of the overall species composition, 8.97% of variation in species composition when cultural species were removed, 9.9% of variation in stenotopic species composition, and 8.23% of variation in ubiquitous species composition (Table 3). None of the edge-related variables were significant for cultural species composition. Similar findings were obtained when singletons were excluded from ubiquitous species.

**Table 3:** Contribution of edge-related variables to variation in species composition in natural areas adjacent to orchards, as explained by partial RDA.

	All species	All min cultural	Stenotopic	Cultural	Ubiquitous
**Full model**					
Conditional	7.39%	7.4%	9.1%	–	2.29% (2.45%)
Constrained (edge-related variables)	8.71%***	8.97%***	9.9%***	–	8.23%** (8.63%***)
**Marginal effects**					
Distance edge	2.43%***	2.49%***	3.39%***	–	1.85% (1.94%)
Edge. env	2.11%*	2.32%**	2.57%**	–	1.95% (1.99%)
Impermeability	1.99%*	2.07%*	2.01%	–	2.21%** (2.28%)*
LUI	2.08%*	1.92%	2.17%	–	1.93% (2.04%)

Significance of terms was assessed as marginal effects. Bold values indicate significant fractions. Values in brackets indicate results for analyses with singletons removed. Blank cells indicate no variables were important. Distance edge—distance from orchard edge. Edge. env—edge-associated change in local environmental variables. Impermeability—impermeability of orchard edge. LUI—land-use intensity.

Values indicate proportion of variance explained (full model) and partial fraction of the variation accounted for by each explanatory term (marginal effects). *p < 0.05; **p < 0.01; ***p < 0.001.

The compositional analyses for all species showed that distance from orchard edge, edge-associated changes in local environmental variables, impermeability, and land-use intensity were significant. When excluding cultural species, distance from orchard edge, edge-associated changes in local environmental variables, and impermeability explained significant components of variation. For stenotopic species composition, distance from orchard edge and edge-associated changes in local environmental variables explained significant components. Only impermeability explained a significant component of variation in ubiquitous species composition. Results were similar when excluding singletons from ubiquitous species.

## Discussion

At present there are 701 UNESCO registered Biosphere Reserves (BRs) around the world^51^. Finding generalities in edge responses is critical to our understanding of species distributions across such a wide array of unique socio-ecological systems. Here we assessed the value of a metacommunity framework for predicting patterns of arthropod assemblages along a natural-orchard interface in a BR, the Kogelberg Biosphere Reserve (KBR). We assessed the role of impermeability of the orchard edge, orchard habitat quality, and edge-induced changes in local environmental conditions for epigaeic arthropod assemblages in adjacent natural habitats. Overall, we found that assemblages in natural habitats were influenced by orchards through mechanisms operating both within the natural habitat and within the adjacent transformed area. Multi-species responses to edges were successfully predicted by using species habitat specificity in conjunction with a metacommunity framework.

Changes in species richness and composition at ecosystem boundaries are a composite of individual species responses, which can be extremely variable^5^. Despite this variation, biodiversity is often considered higher at edges^23^. Two reasons are ascribed to this: (1) spillover of individuals from adjacent ecosystems, and (2) unique species associated with edge habitats^23^. Although we did not find overall species richness to be higher near edges, we found each of these two causes to influence arthropod diversity near orchard edges, but to different degrees depending on species habitat specificity.

Distance to orchard edge was important for cultural species in natural areas, with highest species richness close to the edge. Despite the sharp decline, cultural species were still present at 85 m from the orchard edge. Furthermore, none of the environmental variables (edge related or background environmental heterogeneity) was significant for either cultural species richness or composition in the natural area. This suggests that source-sink dynamics are important for cultural species diversity in non-crop areas near orchard edges. However, spillover of cultural species did not result in higher overall species richness near edges, which contrasts with other studies conducted at the interface of crop and non-crop habitats^52^. A lack of spillover induced higher species richness near edges could result from the orchard not supporting enough cultural species to compensate for species loss (although at the distances studied here we did not observe lower species richness near edges for the other distributional groups), or from the spillover of cultural species not being high enough. As has been found previously in this area^53,54^, many more arthropod species were found in fynbos habitat than orchards, which supports the suggestion that cultural species richness may not be high enough to result in higher overall species richness near edges.

Edge-biased distribution of species can result from differences in vegetation structure and microclimate at edges^55,56^. As predicted, edge-related changes in local environmental variables were more important for stenotopic species composition than for either cultural or ubiquitous species. Edge-associated changes in local environmental variables were related to litter cover (higher litter cover near edges) and vegetation structure (less vegetation cover and lower vegetation height near edges). Some orchards were adjacent to firebreaks. These firebreaks are situated close to the orchard edge, are approximately 10–20 m wide, and mowed to maintain a relatively open vegetation structure, which is contributing to differences in vegetation structure near the orchard edge compared to that further away. Assemblage level responses to edge-associated changes in local environmental variables could be a response to unique local environmental conditions that result from the mixing of orchard and remnant natural area conditions and/or these secondary management interventions. It is also important to note that these results could be confounded by the influence of local vegetation structure on pitfall trapping results^57^. Such an effect would be strongest between habitats that show the strongest difference in vegetation structure.

In addition to edge-related changes in local environmental variables, background environmental heterogeneity was also more important for stenotopic species composition than for the other groups. These results suggest that species sorting is particularly strong for stenotopic species in natural areas. Edge-related changes in local environmental variables was, however, not important for stenotopic species richness. Rather, these changes were related to higher overall species richness, which seemed to be driven by the response of ubiquitous species. Species that avoid transformed areas are more vulnerable to negative effects of fragmentation, while exploiters of these areas remain stable or increase^58^. This could explain why stenotopic species richness here did not show the same response as ubiquitous species richness to changes in local environmental conditions. However, the disparity between species richness and composition suggests that biodiversity change measured as species richness alone can be a weak indicator of ecological impact^8,59^. When environmental change favours some species over others, and immigration and extinction are equal, strong changes in species composition can be associated with little changes in overall species richness^60^.

Management intensity can affect arthropod diversity and abundance in crop habitats^61,62^, which we also found here for arthropods in adjacent non-crop patches. The prediction that management-related variables will be more important for ubiquitous and cultural species than for stenotopic species was upheld by our results, but only in terms of species richness. Structural edge contrast is an important factor determining edge responses^63^, and as predicted, impermeability was more important for ubiquitous species than either stenotopic or cultural species. Importantly however, this was only apparent for changes in species composition.

Agroecosystems are characterised by organisms dispersing and foraging between crop and non-crop ecosystems^9^. Our results support the notion that differences in vulnerability of agricultural vs. natural-area species to management within crop areas can be explained by differences in exposure^20^, which for some species can be a function of biotope contrast. The negative relationship between land-use intensity and species richness suggests that higher intensity orchards are acting as sink habitats (i.e. ecological traps). This impact can be especially important for diversity in adjacent natural patches, as most species sampled here were ubiquitous.

Important limitations of the present study are related to sampling intensity and the use of morphospecies. Arthropods were sampled during periods when they are most active in the study region. The results therefore represent a seasonal snapshot, and longer sampling periods would have allowed us to explore important temporal patterns (e.g. Ref.^10^). Morphospecies are used in diverse biodiversity rich countries with poor arthropod taxonomic resolution, this may affect the results here, especially the total number of species recorded, but is unlikely to change the patterns reported here^64^. In addition, we did not consider potential interactions among the different orchard effects. For example, orchard age can influence the amount of insecticide drift to adjacent natural areas^65,66^, which suggests a potential interaction between orchard impermeability and quality. The lack of interactions in our models, and the wide array of taxa considered, could contribute to the low amount of explained deviance in community composition, as compared to other single taxa studies (e.g. Ref.^28^).

Cross-edge spillover of agriculturally-subsidised species is expected to be most likely under conditions where there is a strong gradient in productivity^9,30^, and can result in changes in ecosystem function^25,30,67^. Excessive local dispersal with strong source-sink relations among different ecosystems can reduce ecosystem functioning by swamping local filters, which would normally favour better-adapted species^68^. Transformed areas can also affect the spread of invasive species and the susceptibility of communities to invasions^69^. To reduce influence of spillover from cultural species on sensitive habitats within farms (e.g. such as riparian areas^70,71^), farmers in the KBR can maintain local buffer strips that are ideally > 85 m wide. Within this strip, management interventions that manipulate vegetation structure to maintain heterogeneous conditions over short distances (e.g. maintaining firebreaks, or clearing alien trees) can promote arthropod diversity^72^. Maintaining orchard habitat quality (by reducing local land-use intensity), will further promote diversity in adjacent non-crop habitats.

### Value for biosphere reserve design and management

Habitat edges informed the early development of BRs^73^, but are not given the same amount of consideration in more recent guidelines (e.g. Ref.^74^). Indeed, assessments of BR zones have viewed these spaces largely from the perspective of island biogeography^75^, reducing landscapes to patches of habitat and non-habitat (e.g. Ref.^76^), and ignoring the complex nature of habitat edges (e.g. Ref.^77–79^, but see Ref.^38^). The results here show that characteristics of the transformed areas are important for assemblages occurring in remaining natural areas, which emphasizes the presence of a gradual boundary between natural-anthropogenic habitats and that the variegated nature of transformed landscapes can contribute in various ways to overall biodiversity^80,81^. As BRs (especially the buffer and transition zones) often represent the interface between natural and transformed areas (e.g. the Los Tuxtlas BR in Mexico, the Białowieża BR in Poland, and the Sakaerat BR in Thailand), emphasis should be placed on understanding the flow of energy and materials (including organisms) across natural-anthropogenic interfaces. The results highlight the value of not viewing BRs as static entities^82^, but rather as ‘open’ reserves in a broader landscape context^83^.

## Supplementary information

Supplementary Appendix 1.

Supplementary Appendix 2.

Supplementary Figure S1.

Supplementary Table S1.

Supplementary Table S2.

Supplementary Table S3.

Supplementary Table S4.

Supplementary Table S5.
