# Supplementary material for: Environmental filtering and spillover explain multi-species edge responses across agricultural boundaries in a biosphere reserve
Source: Sci Rep. 2020 Sep 9;10:14800. doi: 10.1038/s41598-020-71724-1 (PMC7481220; doi:10.1038/s41598-020-71724-1)
Supplement: Supplementary file 1 — Supplementary Appendix 1. [file 41598_2020_71724_MOESM1_ESM.docx]

Appendix 1 Details on measurements of local environmental conditions, impermeability, and local scale land-use intensity (LUI).

1. Measurements of local environmental conditions

Habitat structure was measured in terms of vertical and horizontal vegetation characteristics using similar methods as Parr, Robertson, Biggs and Chown (2004) and Munyai and Foord (2012). At each pitfall trap (i.e. each corner of the 10 x 10 m plot) vertical vegetation height profiles were measured at four points located at 90 degrees apart in a 1.5 m radius centred on each trap. At each point, a 1.5 m pole (1 cm diameter) was held vertically and the total times vegetation came into contact with the pole was noted at 25 cm intervals. Horizontal structure was measured by placing a 1 m^2^ quadrat over each trap and visually estimating the percentage of vegetation cover. Visual estimations were also made of percentage bare ground and litter cover around each trap. To keep visual estimates consistent across sites, photos of quadrats were taken and all estimates were done by the same person with the aid of a visual estimation guide as reference. Measurements of vegetation structure taken at each trap was averaged to obtain a single measure per plot. At each plot, four soil moisture measurements were taken using a soil moisture meter (ZD-05 pH and Moisture Meter). These measurements were averaged for each plot.

Table A1 Variables measured at each plot

| Variable | Description | Source |
| --- | --- | --- |
| Average vertical vegetation structure | Height of vegetation in cm, averaged over each trap | Site measured |
| Average vegetation density | Vegetation density, averaged over each trap | Site measured |
| Average horizontal vegetation structure | Vegetation cover (%), averaged over each trap | Site measured |
| Average ground cover | Ground cover (%), averaged over each trap | Site measured |
| Average rock cover | Rock cover (%), averaged over each trap | Site measured |
| Average litter cover | Litter cover (%), averaged over each trap | Site measured |
| Average soil moisture | Soil moisture (%), averaged over each trap | Site measured |

2. Impermeability

We used a composite measure to describe the amount of contrast between orchards and adjacent fynbos patches that incorporate orchard age, as well as orchard edge (crop to non-crop interface) and tree row orientation in relation to the sun. For each site, the orientation of the nearest crop to non-crop interface was classified as either northern (orchard edge facing from northwest to northeast: 0) or southern (orchard edge facing from south west to south east: 1). For tree row orientation, we classified sites as either north-to-south orientation (0) or east-to-west orientation (1). We used simple averaging to create a composite variable describing the decrease in incoming solar energy within orchard and its nearest edge by summing the z scores of the original variables.

Table A2 Variables included in composite measure of impermeability

| Variable | Description | Source |
| --- | --- | --- |
| Orchard age | Orchard age (years) | Interviews with farmers |
| Edge orientation | Orientation of crop to non-crop interface (northern = 0, southern = 1) | Site measured |
| Tree row orientation | Orientation of tree rows (north-to-south = 0, east-to-west = 1) | Site measured |

3. Local scale land-use intensity

We describe local scale land-use intensity (LUI) using a quantitative, continuous index (similar to Blüthgen et al. (2012, 2016), Hendrickx et al. (2007) and Sosa-Aranda et al. (2018)) based on information gained from farmers, and measurements of understory plant family richness taken for each orchard. We include measures of cover crop management as work has shown that, compared to the absence of cover crops, actively maintaining cover crops can be beneficial for arthropod diversity within orchards (Mailloux et al. 2010, Wan et al. 2014).

The index summarizes different agrochemical inputs and cover crop management, irrespective of the complex relationships present between these factors, and is based on the management information that was available to us and observation in the field. The compound index summarizes standardized intensity of pesticide application (insecticide and fungicide), cover crop management, and fertilization for the period that arthropods were sampled. For insecticide application, we summed the number of seasons that broad spectrum insecticides were applied (0: none, 1: only early in the growing season, 2: early and late in the growing season). We used a similar approach to sum fungicide applications. Cover crop management in these orchards consists of actively sown seed mixes, and the absence of cover crop management is associated with no or very little cover crop. We used the absence of cover crop management (1: no cover crop management, 0: cover crop management present) to represent its influence on arthropod diversity. Within orchards, understory plants were surveyed in the orchard alley within a 1 m^2^ quadrat centred on each pitfall trap. Plants were identified to family level only. We included the inverse of the number of weed families recorded as a measure of habitat homogenization. Fertilization used by farmers was quantified as kg nitrogen per hectare per year. To obtain a measure of local scale LUI, each component was standardized relative to its mean and then summed for each site (Blüthgen et al., 2012).

Table A3 Variables included in composite measure of land-use-intensity in adjacent orchards

| Variable | Description |  |
| --- | --- | --- |
| Insecticide application | Number of seasons that broad spectrum insecticides were applied | Interviews with farmers |
| Fungicide application | Number of seasons that fungicides were applied | Interviews with farmers |
| Absence of cover crop management | Absence of cover crop management (1), or presence (0) | Interviews with farmers |
| Inverse of weed family richness | Inverse of cover-crop family-richness | Site measured |
| Fertilization | Amount of nitrogen (kg) applied per hectare per year | Interviews with farmers |

References

Blüthgen, N., Dormann, C. F., Prati, D., Klaus, V. H., Kleinebecker, T., Hölzel, N., … Weisser, W. W. (2012). A quantitative index of land-use intensity in grasslands: Integrating mowing, grazing and fertilization. *Basic and Applied Ecology*, 13, 207-220.

Blüthgen, N., Simons, N. K., Jung, K., Prati, D., Renner, S. C., Boch, S., … Gossner, M. M. (2016). Land use imperils plant and animal community stability through changes in asynchrony rather than diversity. *Nature Communications,* 7, 10697.

Hendrickx, F., Maelfait, J. -P., van Wingerden, W., Schweiger, O., Speelmans, M., Aviron, S., … Bugter, R. (2007). How landscape structure, land-use intensity and habitat diversity affect components of total arthropod diversity in agricultural landscapes. *Journal of Applied Ecology,* 44, 340-351.

Mailloux, J., Le Bellec, F., Kreiter, S., Tixier, M.-S., & Dubois, P. (2010) Influence of ground cover management on diversity and density of phytoseiid mites (Acari: Phytoseiidae) in Guadeloupean citrus orchards. *Experimental and Applied Acarology*, 52, 275-290.

Munyai, T. C., & Foord, S. H. (2012). Ants on a mountain: spatial, environmental and habitat associations along an altitudinal transect in a centre of endemism. *Journal of Insect Conservation*, 16, 677-695.

Parr, C. L., Robertson, H. G., Biggs, H. C., & Chown, S. L. (2004). Response of African savanna ants to long-term fire regimes. *Journal of Applied Ecology*, 41, 630-642.

Sosa-Aranda, I., del-Val, E., Hernández-Martínez, G., Arroyo-Lambaer, D., Uscanga, A., Boege, K. (2018). Response of lepidopteran herbivore communities to crop management in coffee plantations. *Agriculture, Ecosystems & Environment,* 265, 37-44.

Wan, N.-F., Gu, X.-J., Ji, X.-Y., Jiang, J.-X., Wu, J.-H., Li, B. (2014) Ecological engineering of ground cover vegetation enhances the diversity and stability of peach orchard canopy arthropod communities. *Ecological Engineering*, 70, 175-182
