# Supplementary figures and images for: Environmental filtering and spillover explain multi-species edge responses across agricultural boundaries in a biosphere reserve
Source: Sci Rep. 2020 Sep 9;10:14800. doi: 10.1038/s41598-020-71724-1 (PMC7481220; doi:10.1038/s41598-020-71724-1)

Appendix 2 - Species accumulation curves for all species


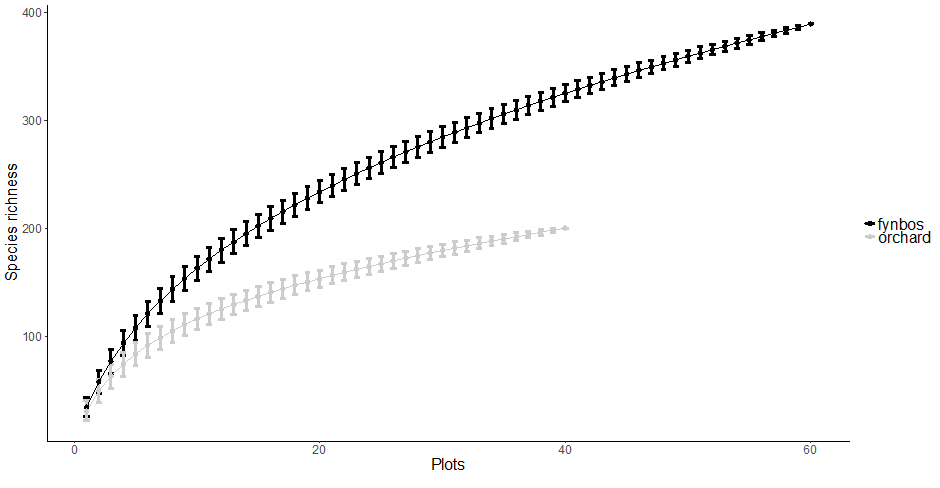

Supplement: Supplementary file 2 — Supplementary Appendix 2. [file 41598_2020_71724_MOESM2_ESM.docx]
