# Supplementary material for: Environmental filtering and spillover explain multi-species edge responses across agricultural boundaries in a biosphere reserve
Source: Sci Rep. 2020 Sep 9;10:14800. doi: 10.1038/s41598-020-71724-1 (PMC7481220; doi:10.1038/s41598-020-71724-1)

Figure S1 Generalized linear mixed model relating predicted species richness of a) all species with cultural species removed, b) ubiquitous species, and c) ubiquitous species with singletons removed to edge related changes in local environmental variables (principal component of DAPC) and d) all species with cultural species removed, e) cultural species, f) ubiquitous species and g) ubiquitous species with singletons removed to land-use intensity (LUI). Spp – species richness.


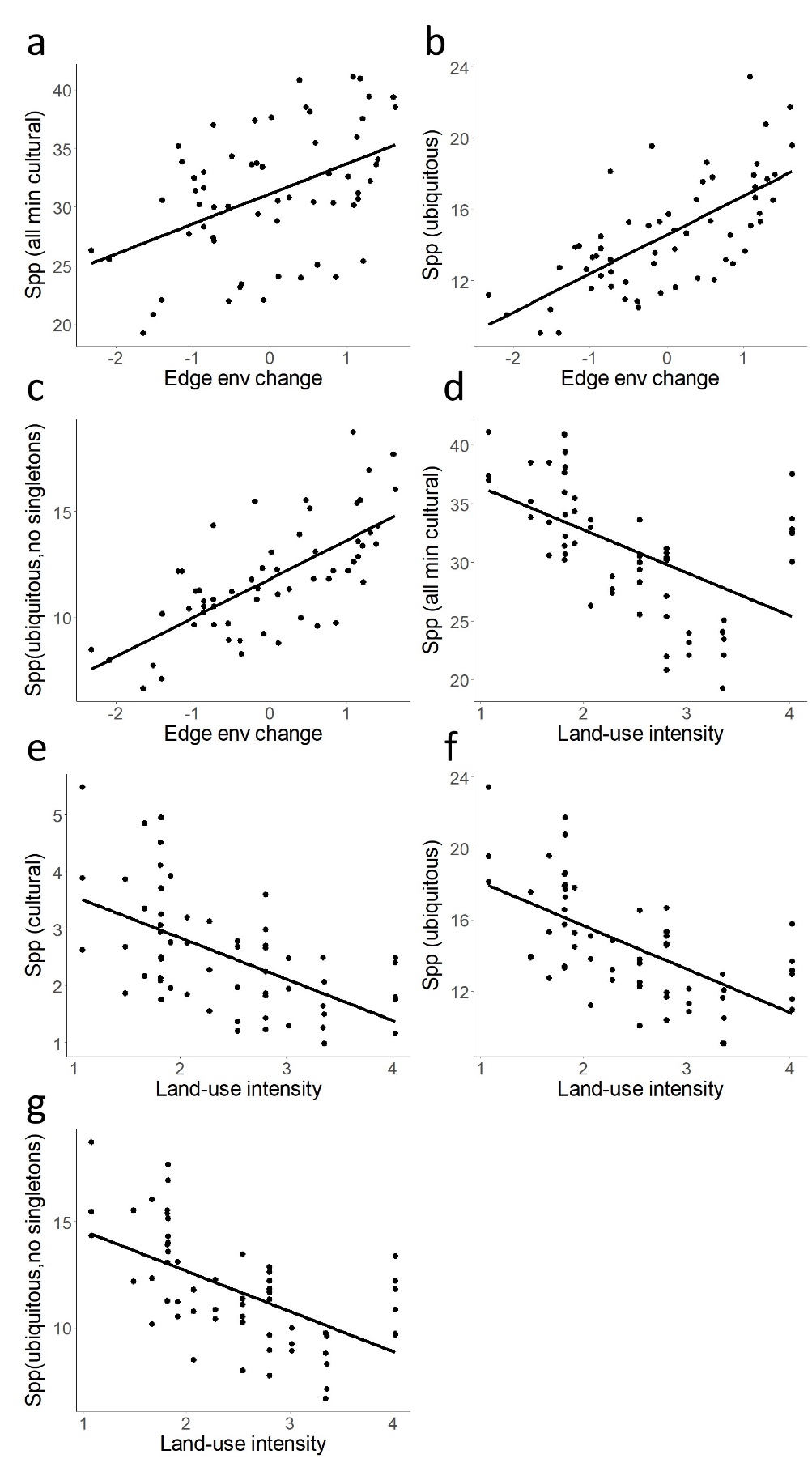

Supplement: Supplementary file 3 — Supplementary Figure S1. [file 41598_2020_71724_MOESM3_ESM.docx]
