# Supplementary material for: Environmental filtering and spillover explain multi-species edge responses across agricultural boundaries in a biosphere reserve
Source: Sci Rep. 2020 Sep 9;10:14800. doi: 10.1038/s41598-020-71724-1 (PMC7481220; doi:10.1038/s41598-020-71724-1)
Supplement: Supplementary file 4 — Supplementary Table S1. [file 41598_2020_71724_MOESM4_ESM.docx]

Table S1 Detailed description of management information grained from farmers. CCM – cover crop management. Pesticide - number of seasons that broad spectrum insecticides were applied (0: none, 1: only early in the growing season, 2: early and late in the growing season), Fungicide - number of seasons that fungicides were applied (0: none, 1: only early in the growing season, 2: early and late in the growing season), Fertilizer - Fertilization used by farmers was quantified as kg nitrogen per hectare per year. Weed families – number of weed families recorded in understory of each orchard.

| Site | Pesticide | Fungicide | Absence of CCM | Fertilizer | Weed families |
| --- | --- | --- | --- | --- | --- |
| 1 | 1 | 2 | 1 | 115 | 5 |
| 2 | 1 | 2 | 1 | 38 | 6 |
| 3 | 1 | 2 | 1 | 114 | 6 |
| 4 | 1 | 2 | 1 | 90.96 | 4 |
| 5 | 1 | 1 | 0 | 83 | 3 |
| 6 | 1 | 1 | 0 | 24 | 4 |
| 7 | 1 | 1 | 0 | 94 | 4 |
| 8 | 0 | 2 | 1 | 109.5 | 4 |
| 9 | 0 | 2 | 1 | 47.05 | 6 |
| 10 | 0 | 2 | 1 | 109.5 | 7 |
| 11 | 2 | 1 | 1 | 109.5 | 10 |
| 12 | 2 | 1 | 1 | 109.5 | 5 |
| 13 | 2 | 0 | 0 | 23.52 | 4 |
| 14 | 2 | 0 | 0 | 0 | 6 |
| 15 | 2 | 0 | 0 | 23.52 | 7 |
| 16 | 2 | 2 | 0 | 94 | 5 |
| 17 | 2 | 2 | 0 | 94 | 5 |
| 18 | 2 | 2 | 0 | 94 | 5 |
| 19 | 2 | 1 | 0 | 24 | 5 |
| 20 | 2 | 1 | 0 | 24 | 5 |
