# Supplementary material for: Environmental filtering and spillover explain multi-species edge responses across agricultural boundaries in a biosphere reserve
Source: Sci Rep. 2020 Sep 9;10:14800. doi: 10.1038/s41598-020-71724-1 (PMC7481220; doi:10.1038/s41598-020-71724-1)
Supplement: Supplementary file 5 — Supplementary Table S2. [file 41598_2020_71724_MOESM5_ESM.docx]

Table S2 List of all insecticides sprayed in orchard blocks which A) only spray broad-spectrum insecticides early in the growing season, B) spray broad-spectrum insecticides early and late in the growing season as well as the major target pests (according to interviews). MB – mealybug species, SI - scale insect species, BFW – banded fruit weevil (Phlyctinus callosus), CM - Codling moth (Cydia pomonella), BW – bollworm (Helicoverpa armigera), TSM – twospotted mite (Tetranychus urticae), WAA – wooly apple aphid (Eriosoma lanigerum), Various – various pests (none specified)

| Active ingredient | Trade name | Target pests | Sprayed early | Sprayed late |
| --- | --- | --- | --- | --- |
| A |  |  |  |  |
| Chlorpyrifos | Chlorpyrifos | MB, SI | x |  |
| Prothiophos | Tokuthion | MB | x |  |
| Novaluron | Rimon | CM, BW | x |  |
| Spinetoram | Delegate | CM, BW, BFW |  | x |
| Thiacloprid | Topstar | CM |  | x |
| Chlorantraniliprole | Altacor | CM, BW | x | x |
| Indoxacarb | Steward | CM, BW, BFW | x | x |
| B |  |  |  |  |
| Chlorfenapyr | Hunter | BFW | x |  |
| Carbaryl | Sevin XLR | BFW | x |  |
| Chlorpyrifos | Chlorpyrifos | MB, TSM | x |  |
| Prothiophos | Tokuthion | MB | x |  |
| Lambda-cyhalothrin | Karate | BW, TSM | x | x |
| Azinphos-methyl | Azinphos | Various | x | x |
| Acetamiprid | Mospilan | CM | x | x |
| Imidacloprid | Confidor | WAA | x | x |
| Chlorantraniliprole | Altacor | CM, BW | x | x |
| Indoxacarb | Steward | TSM, Various | x | x |
| Thiacloprid | Calypso | CM | x | x |
| Novaluron | Rimon | CM, BW | x |  |
| Methoxyfenozid | Runner | CM | x | x |
