# Supplementary material for: Environmental filtering and spillover explain multi-species edge responses across agricultural boundaries in a biosphere reserve
Source: Sci Rep. 2020 Sep 9;10:14800. doi: 10.1038/s41598-020-71724-1 (PMC7481220; doi:10.1038/s41598-020-71724-1)
Supplement: Supplementary file 6 — Supplementary Table S3. [file 41598_2020_71724_MOESM6_ESM.docx]

Table S3 Environmental variables representing background environmental heterogeneity. For more information see van Schalkwyk (2019).

| Description | Grouping | Code | Data type | Data set/source |
| --- | --- | --- | --- | --- |
| Fire frequency in last 10 years | Disturbance | fireFreq_10 | Spatial | CapeNature (2016) |
| Standard deviation of vegetation at 30 m resolution | Disturbance | ageStdev | Spatial | CapeNature (2016) |
| Standard deviation of mean monthly minimum temperature across the year (◦C) | Mesoclimate | stdevMinTemp | Spatial | van Niekerk & Joubert 2011 |
| Average percentage clay | Geology | Ave_Clay | Spatial | ARC-ISCW (2006) |
| Distance (Euclidean) to shale bands | Geology | distShale | spatial | Mucina et al. 2006 |
| Average vegetation height calculated as average touch height | Site characteristic | vegHeightAve | Site measured | Site measured |
| Average moisture (moisture readings per plot average over number of readings) | Site characteristic | moistAve | Site measured | Site measured |

References/data sources

ARC-ISCW (Agricultural Research Council – Institute for Soil, Climate and Water) (2006) Land types of South Africa: Digital map (1:250 000 scale) and soil inventory datasets [vector]

CapeNature (2016) CapeNature Fires – All 2016/17 [Vector] 2016. Available from the Biodiversity GIS website, bgis.sanbi.org

Mucina L, Rutherford MC (2006) The vegetation of South Africa, Lesotho and Swaziland [vector]. Strelitzia 19. South African National Biodiversity Institute, Pretoria

Van Niekerk A, Joubert SJ (2011) Input variable selection for interpolating high-resolution climate surfaces for the Western Cape. Water SA 37: 271-280

van Schalkwyk (2019) Beta diversity across the complementary zones of the Kogelberg Biosphere Reserve. PhD thesis. Stellenbosch University, Stellenbosch.
