# Supplementary material for: Environmental filtering and spillover explain multi-species edge responses across agricultural boundaries in a biosphere reserve
Source: Sci Rep. 2020 Sep 9;10:14800. doi: 10.1038/s41598-020-71724-1 (PMC7481220; doi:10.1038/s41598-020-71724-1)
Supplement: Supplementary file 7 — Supplementary Table S4. [file 41598_2020_71724_MOESM7_ESM.docx]

Table S4 Species and morphospecies sampled in fynbos and orchards, with their total abundances.

| Class | Order | Superfamily | Family | Genus | Species/morphospecies | Abundance |
| --- | --- | --- | --- | --- | --- | --- |
| Arachnida | Araneae | - | Anapidae | Crozetulus | Crozetulus rhodesiensis | 8 |
| Arachnida | Araneae | - | Araneidae | Araneus | Araneus1 | 1 |
| Arachnida | Araneae | - | Caponiidae | Caponia | Caponia capensis | 1 |
| Arachnida | Araneae | - | Clubionidae | Clubiona | Clubiona4 | 2 |
| Arachnida | Araneae | - | Clubionidae | Clubiona | Clubiona4.5 | 8 |
| Arachnida | Araneae | - | Corinnidae | Afroceto | Afroceto capensis | 6 |
| Arachnida | Araneae | - | Corinnidae | Castianeira | Castianeira25.5b | 1 |
| Arachnida | Araneae | - | Corinnidae | Copa | Copa flavoplumosa | 1 |
| Arachnida | Araneae | - | Ctenidae | Ctenus | Ctenus25.4d | 2 |
| Arachnida | Araneae | - | Cyatholipidae | Cyatholipus | Cyatholipus avus | 2 |
| Arachnida | Araneae | - | Cyatholipidae | Cyatholipus | Cyatholipus quadrimaculatus | 21 |
| Arachnida | Araneae | - | Cyatholipidae | Ilisoa | Ilisoa conjugalis | 1 |
| Arachnida | Araneae | - | Cyrtaucheniidae | Ancylotrypa | Ancylotrypa sp1 | 2 |
| Arachnida | Araneae | - | Eutichuridae | Cheiramiona | Cheiramiona ansiae | 1 |
| Arachnida | Araneae | - | Gnaphosidae | Amusia | Amusia cataracta | 7 |
| Arachnida | Araneae | - | Gnaphosidae | Aphantaulax | Aphantaulax inornata | 14 |
| Arachnida | Araneae | - | Gnaphosidae | Asemeshes | Asemeshes sp2 | 3 |
| Arachnida | Araneae | - | Gnaphosidae | Asemesthes | Asemesthes43 | 1 |
| Arachnida | Araneae | - | Gnaphosidae | Camillina | Camillina cordifera | 2 |
| Arachnida | Araneae | - | Gnaphosidae | Trachyzelotes | Trachyzelotes jaxartensis | 1 |
| Arachnida | Araneae | - | Gnaphosidae | Xerophaeus | Xerophaeus capensis | 11 |
| Arachnida | Araneae | - | Gnaphosidae | Zelotes | Zelotes fuligineus | 137 |
| Arachnida | Araneae | - | Hahniidae | Hahnia | Hahnia laticeps | 17 |
| Arachnida | Araneae | - | Linyphiidae | Agyneta | Agyneta habra | 10 |
| Arachnida | Araneae | - | Linyphiidae | Meioneta | Meioneta prosectoides | 9 |
| Arachnida | Araneae | - | Linyphiidae | Mermessus | Mermessus fradeorum | 1 |
| Arachnida | Araneae | - | Linyphiidae | Pelecopsis | Pelecopsis janus | 33 |
| Arachnida | Araneae | - | Lycosidae | Allocosa | Allocosa lawrencei | 101 |
| Arachnida | Araneae | - | Lycosidae | Foveosa | Foveosa foveolata | 8 |
| Arachnida | Araneae | - | Lycosidae | Hogna | Hogna unicolor | 66 |
| Arachnida | Araneae | - | Lycosidae | Minicosa | Minicosa neptuna | 10 |
| Arachnida | Araneae | - | Lycosidae | Pardosa | Pardosa crassipalpis | 8 |
| Arachnida | Araneae | - | Lycosidae | Pardosa | Pardosa12.7 | 6 |
| Arachnida | Araneae | - | Lycosidae | Proevippa | Proevippa biampliata | 116 |
| Arachnida | Araneae | - | Lycosidae | Proevippa | Proevippa12.6g | 2 |
| Arachnida | Araneae | - | Lycosidae | Trabea | Trabea purcelli | 1 |
| Arachnida | Araneae | - | Lycosidae | Trabea | Trabea12.3 | 1 |
| Arachnida | Araneae | - | Migidae | Moggridgea | Moggridgea peringueyi | 5 |
| Arachnida | Araneae | - | Opiliones | - | Opiliones49 | 2 |
| Arachnida | Araneae | - | Opiliones | - | Opiliones49b | 1 |
| Arachnida | Araneae | - | Oxyopidae | Oxyopes | Oxyopes longispinosus | 1 |
| Arachnida | Araneae | - | Philodromidae | Suemus | Suemus punctata | 3 |
| Arachnida | Araneae | - | Philodromidae | Thanatus | Thanatus lamottei | 2 |
| Arachnida | Araneae | - | Pholcidae | Spermophora | Spermophora suurbraak | 1 |
| Arachnida | Araneae | - | Phyxelididae | Malaika | Malaika delicatula | 4 |
| Arachnida | Araneae | - | Phyxelididae | Malaika | Malaika n.sp. | 4 |
| Arachnida | Araneae | - | Pisauridae | Chiasmopes | Chiasmopes lineatus | 1 |
| Arachnida | Araneae | - | Pisauridae | Euprosthenopsis | Euprosthenopsis pulchella | 38 |
| Arachnida | Araneae | - | Pisauridae | Rothus | Rothus aethiopicus | 1 |
| Arachnida | Araneae | - | Prodidomidae | Theuma | Theuma4.5d | 28 |
| Arachnida | Araneae | - | Salticidae | Baryphas | Baryphas ahenus | 2 |
| Arachnida | Araneae | - | Salticidae | Langona | Langona3.5d | 1 |
| Arachnida | Araneae | - | Salticidae | Langona | Langona7.3b | 1 |
| Arachnida | Araneae | - | Salticidae | Massagris | Massagris regina | 6 |
| Arachnida | Araneae | - | Salticidae | Pellenes | Pellenes tharinae | 12 |
| Arachnida | Araneae | - | Salticidae | Rumburak | Rumburak bellus | 20 |
| Arachnida | Araneae | - | Salticidae | Rumburak | Rumburak3.4c | 1 |
| Arachnida | Araneae | - | Salticidae | Thyenula | Thyenula3.4e | 1 |
| Arachnida | Araneae | - | Scytodidae | Scytodes | Scytodes flagellata | 20 |
| Arachnida | Araneae | - | Theridiidae | Euryopis | Euryopis episinoides | 4 |
| Arachnida | Araneae | - | Theridiidae | Euryopis | Euryopis funebris | 1 |
| Arachnida | Araneae | - | Theridiidae | Theridion | Theridion5.7 | 4 |
| Arachnida | Araneae | - | Theridiosomatidae | - | Theridiosomatidae33c | 12 |
| Arachnida | Araneae | - | Thomisidae | Monaeses | Monaeses pustulosus | 1 |
| Arachnida | Araneae | - | Thomisidae | Thomisops | Thomisops sulcatus | 2 |
| Arachnida | Araneae | - | Thomisidae | Xysticus | Xysticus sagittifer | 4 |
| Arachnida | Araneae | - | Zodariidae | Caesetius | Caesetius globicoxis | 1 |
| Arachnida | Araneae | - | Zodariidae | Cydrela | Cydrela25.6 | 2 |
| Arachnida | Araneae | - | Zodariidae | Cydrela | Cydrela42 | 1 |
| Arachnida | Araneae | - | Zodariidae | Cydrela | Cydrela6.7 | 2 |
| Arachnida | Araneae | - | Zodariidae | Cydrela | Cydrela6.7b | 1 |
| Arachnida | Araneae | - | Zodariidae | Diores | Diores simoni | 3 |
| Arachnida | Araneae | - | Zodariidae | Diores | Diores5.2d | 69 |
| Arachnida | Araneae | - | Zodariidae | Heradida | Heradida speculigera | 10 |
| Arachnida | Mesostigmata | - | - | - | Mesostigmata1.6 | 20 |
| Arachnida | Mesostigmata | - | - | - | Mesostigmata1.6c | 11 |
| Arachnida | Mesostigmata | - | - | - | Mesostigmata1.6e | 19 |
| Arachnida | Mesostigmata | - | - | - | Mesostigmata1.6f | 11 |
| Arachnida | Mesostigmata | - | - | - | Mesostigmata1.7c | 2 |
| Arachnida | Mesostigmata | - | - | - | Mesostigmata3.12c | 1 |
| Arachnida | Mesostigmata | - | - | - | Mesostigmata6.3c | 35 |
| Arachnida | Mesostigmata | - | - | - | Mesostigmata6.3d | 74 |
| Arachnida | Mesostigmata | - | - | - | Mesostigmata6.3e | 9 |
| Arachnida | Mesostigmata | - | - | - | Mesostigmata6.3f | 20 |
| Arachnida | Mesostigmata | - | - | - | Mesostigmata6.3h | 22 |
| Arachnida | Mesostigmata | Dermanyssoidea | Laelapidae | - | Mesostigmata1.7 | 2 |
| Arachnida | Mesostigmata | Parasitoidea | Parasitdae | - | Mesostigmata6.3 | 28 |
| Arachnida | Mesostigmata | Parasitoidea | Parasitdae | - | Mesostigmata6.3b | 15 |
| Arachnida | Oribatida | - | - | - | Oribatida1 | 731 |
| Arachnida | Oribatida | - | - | - | Oribatida1.2 | 170 |
| Arachnida | Oribatida | - | - | - | Oribatida1.3 | 5 |
| Arachnida | Oribatida | - | - | - | Oribatida1.3b | 9 |
| Arachnida | Oribatida | - | - | - | Oribatida1.3c | 9 |
| Arachnida | Oribatida | - | - | - | Oribatida1.3e | 22 |
| Arachnida | Oribatida | - | - | - | Oribatida1.4 | 17 |
| Arachnida | Oribatida | - | - | - | Oribatida1.5 | 6 |
| Arachnida | Oribatida | - | - | - | Oribatida1.8 | 5 |
| Arachnida | Oribatida | - | - | - | Oribatida1.9 | 8 |
| Arachnida | Oribatida | - | - | - | Oribatida1.9b | 119 |
| Arachnida | Oribatida | - | - | - | Oribatida1.9c | 7 |
| Arachnida | Oribatida | - | - | - | Oribatida1b | 300 |
| Arachnida | Oribatida | - | - | - | Oribatida2 | 3 |
| Arachnida | Oribatida | - | - | - | Oribatida2.2 | 1 |
| Arachnida | Oribatida | - | - | - | Oribatida2.2b | 22 |
| Arachnida | Oribatida | - | - | - | Oribatida2.6 | 4 |
| Arachnida | Oribatida | - | - | - | Oribatida2.7 | 1 |
| Arachnida | Oribatida | - | - | - | Oribatida2.8 | 2 |
| Arachnida | Oribatida | - | - | - | Oribatida3.10 | 1 |
| Arachnida | Oribatida | - | - | - | Oribatida3.11 | 3 |
| Arachnida | Oribatida | - | - | - | Oribatida3.11b | 2 |
| Arachnida | Oribatida | - | - | - | Oribatida3.11c | 1 |
| Arachnida | Oribatida | - | - | - | Oribatida3.12 | 8 |
| Arachnida | Oribatida | - | - | - | Oribatida3.12b | 13 |
| Arachnida | Oribatida | - | - | - | Oribatida3.2 | 1 |
| Arachnida | Oribatida | - | - | - | Oribatida3.6b | 5 |
| Arachnida | Oribatida | - | - | - | Oribatida3.6d | 3 |
| Arachnida | Oribatida | - | - | - | Oribatida3.7 | 2 |
| Arachnida | Oribatida | - | - | - | Oribatida3.9 | 2 |
| Arachnida | Pseudoscorpiones | - | - | - | pscorp1 | 1 |
| Arachnida | Pseudoscorpiones | - | - | - | pscorp2 | 7 |
| Arachnida | Pseudoscorpiones | - | - | - | pscorp2.5 | 3 |
| Arachnida | Pseudoscorpiones | - | - | - | pscorp2c | 76 |
| Arachnida | Pseudoscorpiones | - | - | - | pscorp4 | 2 |
| Arachnida | Pseudoscorpiones | - | - | - | pscorp5 | 3 |
| Arachnida | Scorpiones | - | - | - | scorpio1 | 1 |
| Arachnida | Scorpiones | - | - | - | scorpio2 | 5 |
| Arachnida | Trombidiformes | - | - | - | Trombidiformes4.2h | 1 |
| Arachnida | Trombidiformes | - | - | - | Trombidiformes4.5 | 25 |
| Arachnida | Trombidiformes | - | - | - | Trombidiformes4.6f | 13 |
| Arachnida | Trombidiformes | - | - | - | Trombidiformes4.6g | 1 |
| Arachnida | Trombidiformes | - | - | - | Trombidiformes4.7b | 45 |
| Arachnida | Trombidiformes | - | - | - | Trombidiformes4.7c | 11 |
| Arachnida | Trombidiformes | - | - | - | Trombidiformes4.7d | 3 |
| Arachnida | Trombidiformes | - | - | - | Trombidiformes4.7f | 29 |
| Arachnida | Trombidiformes | - | - | - | Trombidiformes4.7h | 15 |
| Arachnida | Trombidiformes | - | - | - | Trombidiformes4.7n | 1 |
| Arachnida | Trombidiformes | - | - | - | Trombidiformes4.9 | 29 |
| Arachnida | Trombidiformes | - | - | - | Trombidiformes5 | 2 |
| Arachnida | Trombidiformes | - | - | - | Trombidiformes6.4c | 2 |
| Arachnida | Trombidiformes | - | - | - | Trombidiformes6.4h | 1 |
| Arachnida | Trombidiformes | Bdelloidea | Bdellidae | - | Trombidiformes6.4 | 18 |
| Arachnida | Trombidiformes | Bdelloidea | Bdellidae | - | Trombidiformes6.4b | 5 |
| Arachnida | Trombidiformes | Bdelloidea | Bdellidae | - | Trombidiformes6.4d | 246 |
| Arachnida | Trombidiformes | Bdelloidea | Bdellidae | - | Trombidiformes6.4f | 1 |
| Arachnida | Trombidiformes | Erythraeoidea | - | - | Trombidiformes4.2 | 2 |
| Arachnida | Trombidiformes | Erythraeoidea | - | - | Trombidiformes4.2b | 130 |
| Arachnida | Trombidiformes | Erythraeoidea | - | - | Trombidiformes4.2c | 12 |
| Arachnida | Trombidiformes | Erythraeoidea | - | - | Trombidiformes4.2d | 5 |
| Arachnida | Trombidiformes | Erythraeoidea | - | - | Trombidiformes4.2e | 347 |
| Arachnida | Trombidiformes | Erythraeoidea | - | - | Trombidiformes4.2g | 1 |
| Arachnida | Trombidiformes | Erythraeoidea | - | - | Trombidiformes4.2k | 1 |
| Arachnida | Trombidiformes | Erythraeoidea | - | - | Trombidiformes4.3c | 1 |
| Arachnida | Trombidiformes | Erythraeoidea | - | - | Trombidiformes4.6b | 1 |
| Arachnida | Trombidiformes | Erythraeoidea | - | - | Trombidiformes4.6d | 4 |
| Arachnida | Trombidiformes | Erythraeoidea | - | - | Trombidiformes4.7 | 1 |
| Arachnida | Trombidiformes | Erythraeoidea | - | - | Trombidiformes4.8 | 85 |
| Arachnida | Trombidiformes | Erythraeoidea | - | - | Trombidiformes4.8b | 1 |
| Arachnida | Trombidiformes | Erythraeoidea | - | - | Trombidiformes4.8c | 7 |
| Arachnida | Trombidiformes | Erythraeoidea | - | - | Trombidiformes4.9c | 4 |
| Arachnida | Trombidiformes | Erythraeoidea | - | - | Trombidiformes4.9d | 8 |
| Arachnida | Trombidiformes | Erythraeoidea | - | - | Trombidiformes4.9g | 1 |
| Arachnida | Trombidiformes | Trombidioidea | Trombidiidae | - | Trombidiformes4.5b | 1 |
| Arachnida | Trombidiformes | Trombidioidea | Trombidiidae | - | Trombidiformes4.5c | 30 |
| Arachnida | Trombidiformes | Trombidioidea | Trombidiidae | - | Trombidiformes4.5d | 6 |
| Arachnida | Trombidiformes | Trombidioidea | Trombidiidae | - | Trombidiformes4.5e | 3 |
| Arachnida | Trombidiformes | Trombidioidea | Trombidiidae | - | Trombidiformes4.5f | 1 |
| Chilopoda | Lithobiomorpha | - | - | - | Lithobiomorpha3 | 22 |
| Chilopoda | Lithobiomorpha | - | - | - | Lithobiomorpha4 | 1 |
| Chilopoda | Lithobiomorpha | - | - | - | Lithobiomorpha4.2 | 5 |
| Diplopoda | - | - | - | - | Diplopoda1 | 63 |
| Diplopoda | - | - | - | - | Diplopoda2 | 44 |
| Diplopoda | - | - | - | - | Diplopoda3 | 5 |
| Diplopoda | - | - | - | - | Diplopoda4 | 2 |
| Diplopoda | Polydemida | - | - | - | Polydesmidae3.2 | 43 |
| Insecta | Archeognatha | - | Meinertellidae | - | Meinertellidae1 | 20 |
| Insecta | Archeognatha | - | Meinertellidae | - | Meinertellidae3 | 12 |
| Insecta | Blattodea | - | - | - | Blaberidae2.2 | 3 |
| Insecta | Blattodea | - | - | - | Blaberidae2.7 | 2 |
| Insecta | Blattodea | - | - | - | Blatellidae1.14 | 1 |
| Insecta | Blattodea | - | - | - | Blattodea1 | 2 |
| Insecta | Blattodea | - | - | - | Blattodea1.5b | 1 |
| Insecta | Blattodea | - | - | - | Blattodea1.8 | 1 |
| Insecta | Blattodea | - | Blatella | - | Blatella germanica | 1 |
| Insecta | Blattodea | - | Saltoblatella | - | Saltoblatella montistabularis | 1 |
| Insecta | Blattodea | - | Temnopteryx | - | Temnopteryx phalerata | 1 |
| Insecta | Blattodea | - | Temnopteryx | - | Temnopteryx1.10 | 1 |
| Insecta | Blattodea | - | Temnopteryx | - | Temnopteryx1.5 | 1 |
| Insecta | Coleoptera | - | - | - | chryso10 | 1 |
| Insecta | Coleoptera | - | - | - | chryso5.7 | 1 |
| Insecta | Coleoptera | - | - | - | chryso5.8 | 2 |
| Insecta | Coleoptera | - | - | - | chryso6 | 1 |
| Insecta | Coleoptera | - | - | - | chryso6.2 | 3 |
| Insecta | Coleoptera | - | Bostrychidae | - | Bostrychidae2 | 18 |
| Insecta | Coleoptera | - | Geotrupidae | - | Geotrupidae5 | 3 |
| Insecta | Coleoptera | - | Geotrupidae | - | Geotrupidae5.2 | 1 |
| Insecta | Coleoptera | - | Melolonthinae | - | Melolonthinae3b | 1 |
| Insecta | Coleoptera | - | Omaliinae | - | Omaliinae3.3c | 4 |
| Insecta | Coleoptera | - | Scarabaeinae | - | Scarabaeinae1.2 | 12 |
| Insecta | Coleoptera | - | Scarabaeinae | - | Scarabaeinae1.2b | 3 |
| Insecta | Coleoptera | - | Scarabaeinae | - | Scarabaeinae3 | 1 |
| Insecta | Coleoptera | - | Scarabaeinae | - | Scarabaeinae6 | 6 |
| Insecta | Coleoptera | - | Staphylinidae | - | Aleocharinae3 | 31 |
| Insecta | Coleoptera | - | Staphylinidae | - | Aleocharinae3b | 12 |
| Insecta | Coleoptera | - | Staphylinidae | - | Aleocharinae5.2e | 1 |
| Insecta | Coleoptera | - | Staphylinidae | - | Oxytelinae3.2 | 7 |
| Insecta | Coleoptera | - | Staphylinidae | - | Oxytelinae3.2b | 1 |
| Insecta | Coleoptera | - | Staphylinidae | - | Oxytelinae3.2c | 1 |
| Insecta | Coleoptera | - | Staphylinidae | - | Oxytelinae3.4 | 1 |
| Insecta | Coleoptera | - | Staphylinidae | - | Oxytelinae3c | 3 |
| Insecta | Coleoptera | - | Staphylinidae | - | Paederinae5.2f | 2 |
| Insecta | Coleoptera | - | Staphylinidae | - | Staphylininae5 | 3 |
| Insecta | Coleoptera | - | Staphylinidae | - | Staphylininae5.2 | 15 |
| Insecta | Coleoptera | - | Trogidae | Trox | Trox1 | 6 |
| Insecta | Coleoptera | - | Trogidae | Trox | Trox2 | 1 |
| Insecta | Coleoptera | Caraboidea | Carabidae | - | Carabidae2 | 28 |
| Insecta | Coleoptera | Caraboidea | Carabidae | - | Carabidae2.2 | 9 |
| Insecta | Coleoptera | Caraboidea | Carabidae | - | Carabidae2.3 | 52 |
| Insecta | Coleoptera | Caraboidea | Carabidae | - | Carabidae2.4b | 251 |
| Insecta | Coleoptera | Caraboidea | Carabidae | - | Carabidae2.5 | 6 |
| Insecta | Coleoptera | Caraboidea | Carabidae | - | Carabidae2.5b | 1 |
| Insecta | Coleoptera | Caraboidea | Carabidae | - | Carabidae4 | 1 |
| Insecta | Coleoptera | Caraboidea | Carabidae | - | Carabidae4.2 | 120 |
| Insecta | Coleoptera | Caraboidea | Carabidae | Thermophilum | T.decemguttatum | 17 |
| Insecta | Coleoptera | Caraboidea | Cicindelidae | - | Cicindelidae1 | 1 |
| Insecta | Coleoptera | Chrysomeloidea | Chrysomelidae | - | beetle4 | 1 |
| Insecta | Coleoptera | Chrysomeloidea | Chrysomelidae | - | beetle7 | 1 |
| Insecta | Coleoptera | Chrysomeloidea | Chrysomelidae | - | Eumolpinae4.3 | 1 |
| Insecta | Coleoptera | Chrysomeloidea | Chrysomelidae | - | Eumolpinae7 | 4 |
| Insecta | Coleoptera | Cucujoidea | - | - | tene9 | 1 |
| Insecta | Coleoptera | Cucujoidea | Alticinae | - | Alticinae1.2 | 7 |
| Insecta | Coleoptera | Cucujoidea | Ciidae | - | Ciidae9 | 2 |
| Insecta | Coleoptera | Cucujoidea | Coccinellidae | - | Coccinellidae3b | 1 |
| Insecta | Coleoptera | Cucujoidea | Coccinellidae | - | Coccinellidae4 | 1 |
| Insecta | Coleoptera | Cucujoidea | Corylophidae | - | Corylophidae4 | 209 |
| Insecta | Coleoptera | Cucujoidea | Cryptophagidae | - | chryso5.5 | 1 |
| Insecta | Coleoptera | Cucujoidea | Cryptophagidae | - | Cryptophagidae4.2 | 3 |
| Insecta | Coleoptera | Cucujoidea | Elateridae | - | elater8c | 2 |
| Insecta | Coleoptera | Cucujoidea | Nitidulidae | Cybocephalus | Cybocephalus1 | 3 |
| Insecta | Coleoptera | Cucujoidea | Nitidulidae | Lasiodactylus | Lasiodactylus1 | 12 |
| Insecta | Coleoptera | Cucujoidea | Nitidulidae | Lasiodactylus | Lasiodactylus9 | 71 |
| Insecta | Coleoptera | Cucujoidea | Tenebrionidae | - | Anthicidae1 | 20 |
| Insecta | Coleoptera | Cucujoidea | Tenebrionidae | - | Anthicidae2 | 2 |
| Insecta | Coleoptera | Cucujoidea | Tenebrionidae | - | Anthicidae3 | 1 |
| Insecta | Coleoptera | Cucujoidea | Tenebrionidae | - | chryso4 | 1 |
| Insecta | Coleoptera | Cucujoidea | Tenebrionidae | - | chryso5.3 | 18 |
| Insecta | Coleoptera | Cucujoidea | Tenebrionidae | - | tene3.2 | 9 |
| Insecta | Coleoptera | Cucujoidea | Tenebrionidae | - | tene3.4 | 3 |
| Insecta | Coleoptera | Cucujoidea | Tenebrionidae | - | tene3b | 2 |
| Insecta | Coleoptera | Cucujoidea | Tenebrionidae | - | tene5 | 15 |
| Insecta | Coleoptera | Cucujoidea | Tenebrionidae | - | tene6 | 18 |
| Insecta | Coleoptera | Cucujoidea | Tenebrionidae | - | tene6.2 | 1 |
| Insecta | Coleoptera | Cucujoidea | Tenebrionidae | Gonocephalum | tene3 | 941 |
| Insecta | Coleoptera | Curculionoidea | Curculionidae | - | weevil1.2c | 1 |
| Insecta | Coleoptera | Curculionoidea | Curculionidae | - | weevil2.2 | 1 |
| Insecta | Coleoptera | Curculionoidea | Curculionidae | - | weevil2.2b | 1 |
| Insecta | Coleoptera | Curculionoidea | Curculionidae | - | weevil2.3 | 5 |
| Insecta | Coleoptera | Curculionoidea | Curculionidae | - | weevil2b | 8 |
| Insecta | Coleoptera | Curculionoidea | Curculionidae | - | weevil2c | 5 |
| Insecta | Coleoptera | Curculionoidea | Curculionidae | - | weevil3 | 1 |
| Insecta | Coleoptera | Curculionoidea | Curculionidae | - | weevil3.3 | 1 |
| Insecta | Coleoptera | Curculionoidea | Curculionidae | - | weevil4 | 1 |
| Insecta | Coleoptera | Curculionoidea | Curculionidae | - | weevil7.2b | 2 |
| Insecta | Coleoptera | Curculionoidea | Curculionidae | - | weevil7b | 1 |
| Insecta | Coleoptera | Curculionoidea | Curculionidae | - | weevil8b | 8 |
| Insecta | Coleoptera | Curculionoidea | Curculionidae | - | weevil8c | 6 |
| Insecta | Coleoptera | Curculionoidea | Curculionidae | - | weevil8d | 1 |
| Insecta | Coleoptera | Curculionoidea | Curculionidae | - | weevil8e | 1 |
| Insecta | Coleoptera | Dermestoidea | Dermestidae | - | Dermestidae2 | 1 |
| Insecta | Coleoptera | Elateroidea | Elateridae | - | elater5 | 1 |
| Insecta | Coleoptera | Elateroidea | Elateridae | - | elater6 | 4 |
| Insecta | Coleoptera | Elateroidea | Elateridae | - | elater7 | 1 |
| Insecta | Coleoptera | Elateroidea | Elateridae | - | elater8 | 1 |
| Insecta | Coleoptera | Histeroidea | Histeridae | - | Histeridae2.2 | 21 |
| Insecta | Coleoptera | Histeroidea | Histeridae | - | Histeridae2.3b | 100 |
| Insecta | Coleoptera | Lymexyloidea | Lymexylidae | Melittomma | Melittomma6 | 1 |
| Insecta | Coleoptera | Staphylinoidea | Pselaphidae | - | Pselaphidae5.13 | 2 |
| Insecta | Coleoptera | Staphylinoidea | Pselaphidae | - | Pselaphidae5.2d | 29 |
| Insecta | Coleoptera | Staphylinoidea | Pselaphidae | - | Pselaphidae5.2e | 5 |
| Insecta | Coleoptera | Staphylinoidea | Scydmaenidae | - | Scydmaenidae5.2 | 52 |
| Insecta | Coleoptera | Staphylinoidea | Silphidae | - | Silphidae3.3 | 1 |
| Insecta | Coleoptera | Staphylinoidea | Staphylinidae | - | Paederinae5.3 | 2 |
| Insecta | Coleoptera | Staphylinoidea | Staphylinidae | - | Tachyporinae1 | 16 |
| Insecta | Coleoptera | Staphylinoidea | Staphylinidae | - | Tachyporinae1.2b | 2 |
| Insecta | Coleoptera | Tenebrionoidea | - | - | tene8.2 | 8 |
| Insecta | Coleoptera | Tenebrionoidea | Mordellidae | - | chryso5.14b | 1 |
| Insecta | Coleoptera | Tenebrionoidea | Mordellidae | - | mordeli3 | 1 |
| Insecta | Coleoptera | Tenebrionoidea | Mordellidae | - | mordeli4 | 3 |
| Insecta | Coleoptera | Tenebrionoidea | Mycetophagidae | - | chryso5 | 1 |
| Insecta | Coleoptera | Tenebrionoidea | Mycetophagidae | - | tene4.3 | 2 |
| Insecta | Dermaptera | - | - | - | Forficulidae1.1 | 3 |
| Insecta | Dermaptera | - | - | - | Forficulidae2 | 1 |
| Insecta | Dermaptera | - | - | - | Forficulidae2c | 3 |
| Insecta | Dermaptera | - | Forficulidae | - | Forficulidae2.2 | 64 |
| Insecta | Hemiptera | - | - | - | hetero1d | 2 |
| Insecta | Hemiptera | - | - | - | hetero3.4b | 1 |
| Insecta | Hemiptera | - | - | - | hetero3.6 | 1 |
| Insecta | Hemiptera | - | - | - | hetero6.5 | 1 |
| Insecta | Hemiptera | - | - | - | hetero8 | 2 |
| Insecta | Hemiptera | - | - | - | hetero9.3 | 1 |
| Insecta | Hemiptera | - | - | - | hetero9.4 | 2 |
| Insecta | Hemiptera | - | - | - | lhop3.3b | 1 |
| Insecta | Hemiptera | - | - | - | lyg1 | 31 |
| Insecta | Hemiptera | - | - | - | pyrrh2.6 | 1 |
| Insecta | Hemiptera | - | - | - | Reduvidae4 | 3 |
| Insecta | Hemiptera | - | Cicadellidae | - | rhop3.2d | 1 |
| Insecta | Hemiptera | - | Cicadellidae | - | rhop5.5 | 1 |
| Insecta | Hemiptera | - | Lygaeidae | - | hetero6.6 | 35 |
| Insecta | Hemiptera | - | Pentatomidae | - | scutel2 | 1 |
| Insecta | Hemiptera | Aphidoidea | - | - | aphid1 | 63 |
| Insecta | Hemiptera | Aphidoidea | - | - | aphid3 | 101 |
| Insecta | Hemiptera | Aphidoidea | - | - | aphid4 | 2 |
| Insecta | Hemiptera | Aphidoidea | - | - | aphid4.2 | 29 |
| Insecta | Hemiptera | Aphidoidea | - | - | aphid4.3 | 4 |
| Insecta | Hemiptera | Aphidoidea | - | - | aphid5.2 | 66 |
| Insecta | Hemiptera | Aphidoidea | - | - | aphid5.4 | 59 |
| Insecta | Hemiptera | Aphidoidea | - | - | aphid5.4b | 48 |
| Insecta | Hemiptera | Aphidoidea | - | - | aphid5b | 29 |
| Insecta | Hemiptera | Aphidoidea | - | - | aphid6.2b | 3 |
| Insecta | Hemiptera | Aphidoidea | - | - | aphid6.2c | 2 |
| Insecta | Hemiptera | Aphidoidea | - | - | aphid6.3 | 25 |
| Insecta | Hemiptera | Aphidoidea | - | - | aphid6b | 9 |
| Insecta | Hemiptera | Aphidoidea | - | - | aphid8.2 | 26 |
| Insecta | Hemiptera | Aphidoidea | - | - | aphid8b | 7 |
| Insecta | Hemiptera | Aphidoidea | - | - | aphid9 | 29 |
| Insecta | Hemiptera | Fulgoroidea | - | - | issi6 | 1 |
| Insecta | Hemiptera | Fulgoroidea | - | - | issi7 | 1 |
| Insecta | Hemiptera | Fulgoroidea | - | - | lhop1 | 1 |
| Insecta | Hemiptera | Fulgoroidea | - | - | lhop11.3 | 1 |
| Insecta | Hemiptera | Fulgoroidea | - | - | lhop14.4 | 1 |
| Insecta | Hemiptera | Fulgoroidea | - | - | lhop15 | 1 |
| Insecta | Hemiptera | Fulgoroidea | - | - | lhop16 | 19 |
| Insecta | Hemiptera | Fulgoroidea | - | - | lhop19 | 3 |
| Insecta | Hemiptera | Fulgoroidea | - | - | lhop19c | 1 |
| Insecta | Hemiptera | Fulgoroidea | - | - | lhop21 | 1 |
| Insecta | Hemiptera | Fulgoroidea | - | - | lhop8 | 2 |
| Insecta | Hemiptera | Fulgoroidea | - | - | lhop8.4 | 1 |
| Insecta | Hemiptera | Lygaeoidea | Blissidae | - | hetero3.5 | 1 |
| Insecta | Hemiptera | Lygaeoidea | Lygaeidae | - | hetero3 | 2 |
| Insecta | Hemiptera | Lygaeoidea | Lygaeidae | - | pyrrh2 | 12 |
| Insecta | Hemiptera | Lygaeoidea | Lygaeidae | - | pyrrh2.2 | 53 |
| Insecta | Hemiptera | Lygaeoidea | Lygaeidae | - | pyrrh2.4 | 8 |
| Insecta | Hemiptera | Lygaeoidea | Lygaeidae | - | pyrrh2.5 | 1 |
| Insecta | Hemiptera | Lygaeoidea | Lygaeidae | - | pyrrh3.3 | 3 |
| Insecta | Hemiptera | Lygaeoidea | Psamminae | - | pyrrh5 | 1 |
| Insecta | Hemiptera | Lygaeoidea | Psamminae | Psammium | Psammium1 | 1 |
| Insecta | Hemiptera | Pentatomoidea | Cydnidae | - | cydni1 | 60 |
| Insecta | Hemiptera | Pentatomoidea | Cydnidae | - | cydni3 | 8 |
| Insecta | Hemiptera | Pentatomoidea | Cydnidae | - | cydni3.2 | 2 |
| Insecta | Hemiptera | Pentatomoidea | Cydnidae | - | cydni3b | 2 |
| Insecta | Hemiptera | Pyrrhocoroidea | Pyrrhocoridae | - | pyrrh4 | 28 |
| Insecta | Hymenoptera | Formicoidea | formicidae | anoplolepis | anoplolepis1.4 | 1031 |
| Insecta | Hymenoptera | Formicoidea | formicidae | Brachymyrmex | Brachymyrmex5.2 | 18 |
| Insecta | Hymenoptera | Formicoidea | formicidae | Brachymyrmex | Brachymyrmex5.2c | 97 |
| Insecta | Hymenoptera | Formicoidea | formicidae | camponotus | camponotus1 | 22 |
| Insecta | Hymenoptera | Formicoidea | formicidae | camponotus | camponotus1.4g | 26 |
| Insecta | Hymenoptera | Formicoidea | formicidae | camponotus | camponotus11.3 | 1 |
| Insecta | Hymenoptera | Formicoidea | formicidae | camponotus | camponotus16 | 1 |
| Insecta | Hymenoptera | Formicoidea | formicidae | camponotus | camponotus1d | 15 |
| Insecta | Hymenoptera | Formicoidea | formicidae | camponotus | camponotus1e | 1 |
| Insecta | Hymenoptera | Formicoidea | formicidae | camponotus | camponotus1g | 89 |
| Insecta | Hymenoptera | Formicoidea | formicidae | camponotus | camponotus7 | 20 |
| Insecta | Hymenoptera | Formicoidea | formicidae | camponotus | camponotus7.2 | 2 |
| Insecta | Hymenoptera | Formicoidea | formicidae | camponotus | camponotus7.3 | 6 |
| Insecta | Hymenoptera | Formicoidea | formicidae | camponotus | camponotus7b | 24 |
| Insecta | Hymenoptera | Formicoidea | formicidae | Cerapachys | Cerapachys8 | 2 |
| Insecta | Hymenoptera | Formicoidea | formicidae | Cerapachys | Cerapachys8.2 | 1 |
| Insecta | Hymenoptera | Formicoidea | formicidae | Crematogaster | Crematogaster3 | 15 |
| Insecta | Hymenoptera | Formicoidea | formicidae | Crematogaster | Crematogaster3.2 | 15 |
| Insecta | Hymenoptera | Formicoidea | formicidae | Dorylus | Dorylus14b | 14 |
| Insecta | Hymenoptera | Formicoidea | formicidae | Hypoponera | Hypoponera13 | 1 |
| Insecta | Hymenoptera | Formicoidea | formicidae | Hypoponera | Hypoponera15 | 2 |
| Insecta | Hymenoptera | Formicoidea | formicidae | lepisiota | lepisiota1.2 | 1943 |
| Insecta | Hymenoptera | Formicoidea | formicidae | Leptogenys | Leptogenys13b | 1 |
| Insecta | Hymenoptera | Formicoidea | formicidae | Linepithema | Linepithema12 | 74 |
| Insecta | Hymenoptera | Formicoidea | formicidae | Meranoplus | Meranoplus2.5 | 501 |
| Insecta | Hymenoptera | Formicoidea | formicidae | Monomorium | Monomorium2.2b | 527 |
| Insecta | Hymenoptera | Formicoidea | formicidae | Monomorium | Monomorium2.2c | 131 |
| Insecta | Hymenoptera | Formicoidea | formicidae | Monomorium | Monomorium2.2e | 14 |
| Insecta | Hymenoptera | Formicoidea | formicidae | Monomorium | Monomorium2.2j | 1 |
| Insecta | Hymenoptera | Formicoidea | formicidae | Monomorium | Monomorium4.3 | 26 |
| Insecta | Hymenoptera | Formicoidea | formicidae | Myrmicaria | Myrmicaria2 | 12 |
| Insecta | Hymenoptera | Formicoidea | formicidae | Ocymyrmex | Ocymyrmex2.6d | 76 |
| Insecta | Hymenoptera | Formicoidea | formicidae | Oligomyrmex | Oligomyrmex2.2h | 6 |
| Insecta | Hymenoptera | Formicoidea | formicidae | Pheidole | Pheidole2.2 | 5872 |
| Insecta | Hymenoptera | Formicoidea | formicidae | Pheidole | Pheidole2.2g | 885 |
| Insecta | Hymenoptera | Formicoidea | formicidae | Pheidole | Pheidole4 | 3 |
| Insecta | Hymenoptera | Formicoidea | formicidae | Plagiolepis | Plagiolepis1.5 | 4 |
| Insecta | Hymenoptera | Formicoidea | formicidae | Probolomyrmex | Probolomyrmex14 | 12 |
| Insecta | Hymenoptera | Formicoidea | formicidae | Rhoptromyrmex | Rhoptromyrmex2.5b | 2 |
| Insecta | Hymenoptera | Formicoidea | formicidae | Rhoptromyrmex | Rhoptromyrmex2.6e | 1 |
| Insecta | Hymenoptera | Formicoidea | formicidae | Solenopsis | Solenopsis4.6 | 311 |
| Insecta | Hymenoptera | Formicoidea | formicidae | Solenopsis | Solenopsis4.6d | 135 |
| Insecta | Hymenoptera | Formicoidea | formicidae | Tapinoma | Tapinoma5.2b | 81 |
| Insecta | Hymenoptera | Formicoidea | formicidae | Tetramorium | Tetramorium2.3 | 130 |
| Insecta | Hymenoptera | Formicoidea | formicidae | Tetramorium | Tetramorium2.3b | 35 |
| Insecta | Hymenoptera | Formicoidea | formicidae | Tetramorium | Tetramorium2.3c | 59 |
| Insecta | Hymenoptera | Formicoidea | formicidae | Tetramorium | Tetramorium2.3d | 5 |
| Insecta | Hymenoptera | Formicoidea | formicidae | Tetramorium | Tetramorium2.4 | 65 |
| Insecta | Hymenoptera | Formicoidea | formicidae | Tetramorium | Tetramorium2.4b | 292 |
| Insecta | Hymenoptera | Formicoidea | formicidae | Tetramorium | Tetramorium2.4c | 9 |
| Insecta | Hymenoptera | Formicoidea | formicidae | Tetramorium | Tetramorium2.4d | 79 |
| Insecta | Hymenoptera | Formicoidea | formicidae | Tetramorium | Tetramorium2.6 | 1 |
| Insecta | Hymenoptera | Formicoidea | formicidae | Tetramorium | Tetramorium2.6b | 4 |
| Insecta | Hymenoptera | Formicoidea | formicidae | Tetramorium | Tetramorium2.6c | 356 |
| Insecta | Hymenoptera | Formicoidea | formicidae | Tetramorium | Tetramorium2.7 | 4 |
| Insecta | Hymenoptera | Formicoidea | formicidae | Tetraponera | Tetraponera2.6f | 1 |
| Insecta | Orthoptera | - | - | - | Gryllidae6 | 3 |
| Insecta | Orthoptera | - | - | - | Orthoptera4.3 | 2 |
| Insecta | Orthoptera | - | - | - | Orthoptera4.4 | 1 |
| Insecta | Orthoptera | - | Anostostomatidae | Henicus | Henicus2 | 13 |
| Insecta | Orthoptera | - | Anostostomatidae | Henicus | Henicus4b | 1 |
| Insecta | Orthoptera | - | Anostostomatidae | Henicus | Henicus4c | 2 |
| Insecta | Orthoptera | - | Cophogryllus | - | Cophogryllus3 | 12 |
| Insecta | Orthoptera | - | Cophogryllus | - | Cophogryllus3b | 11 |
| Insecta | Orthoptera | - | Cophogryllus | - | Cophogryllus4 | 1 |
| Insecta | Orthoptera | - | Gryllacrididae | Eremus | Eremus5 | 1 |
| Insecta | Orthoptera | - | Gryllidae | - | Gryllidae1 | 43 |
| Insecta | Orthoptera | - | Gryllidae | Cophogryllus | Cophogryllus1 | 17 |
| Insecta | Orthoptera | - | Gryllus | - | Gryllus6.3 | 21 |
| Insecta | Orthoptera | - | Lentulidae | - | Lentulidae2.2 | 1 |
| Insecta | Orthoptera | - | Pamphagidae | - | Pamphagidae6.3 | 1 |
| Insecta | Orthoptera | - | Tetrigidae | - | Tetrigidae1.2 | 1 |
| Insecta | Pscocoptera | - | Liposcelidae | - | Liposcelidae1 | 1 |
| Insecta | Pscocoptera | - | Liposcelidae | - | Liposcelidae2 | 1 |
| Insecta | Psocoptera | - | - | - | aphid5.4c | 14 |
| Insecta | Thysanura | - | Ctenolepisma | - | Ctenolepisma1 | 19 |
| Insecta | Thysanura | - | Ctenolepisma | - | Ctenolepisma2 | 13 |
| Malacostraca | Amphipoda | - | - | - | amphi1 | 73 |
| Malacostraca | Isopoda | - | - | - | Isopoda1 | 7 |
| Malacostraca | Isopoda | - | - | - | Isopoda2.2 | 12 |
| Malacostraca | Isopoda | - | - | - | Isopoda5 | 4 |
| Malacostraca | Isopoda | - | - | - | Isopoda5b | 2 |
| Malacostraca | Isopoda | - | - | - | Pseudococcidae4 | 4 |
| Malacostraca | Isopoda | - | Armadillidiidae | - | Armadillidiidae3 | 8 |
