# Supplementary material for: Environmental filtering and spillover explain multi-species edge responses across agricultural boundaries in a biosphere reserve
Source: Sci Rep. 2020 Sep 9;10:14800. doi: 10.1038/s41598-020-71724-1 (PMC7481220; doi:10.1038/s41598-020-71724-1)
Supplement: Supplementary file 8 — Supplementary Table S5. [file 41598_2020_71724_MOESM8_ESM.docx]

Table S5 – Results of permutation test testing for the effect of background environmental variables on arthropod species composition in fynbos. Significant tests were followed by a forward selection procedure. This was repeated for all, all excluding cultural, stenotopic, cultural and ubiquitous species. fireFreq_10 - fire frequency in last 10 years. distShale - distance to shale bands. moistAve - average soil moisture. vegHeightAve - average vegetation height. Values in brackets are for analyses with singletons removed. Empty cells – no variables selected

|  | All species | All min cultural | Stenotopic | Cultural | Ubiquitous |
| --- | --- | --- | --- | --- | --- |
| F | 1.22*** | 1.23*** | 1.43*** | 1.14 | 1.09*  (1.10*) |
| Variables selected by forward selection | fireFreq_10, distShale, moistAve | fireFreq_10, distShale, vegHeightAve | fireFreq_10, distShale, moistAve |  | fireFreq_10  (fireFreq_10) |
| Values represent F-values  * p < 0.05, ** p < 0.01, *** p < 0.001 | | | |  |  |
